# Hybrid off-grid energy systems optimal sizing with integrated hydrogen storage based on deterministic balance approach

**DOI:** 10.1038/s41598-024-55631-3

**Published:** 2024-03-22

**Authors:** Alaa Selim, Mohamed El-shimy, Ghada Amer, Ilham Ihoume, Hasan Masrur, Josep M. Guerrero

**Affiliations:** 1 Electrical and Computer Engineering Department, University of Connecticut, Storrs, Connecticut USA; 2 Electrical Power and Machines Dept., Faculty of Engineering, Ain Shams University, Cairo, Egypt; 3 University of New South Wales, Sydney, NSW 2052 Australia; 4 Electrical Power and Machines Dept., Faculty of Engineering, Benha University, Benha, Egypt; 5 Solar Energy and Environment Laboratory, Mohammed V University in Rabat, Rabat, Morocco; 6 Interdisciplinary Research Center of Smart Mobility and Logistics, King Fahd University of Petroleum and Minerals, Dhahran, 31261 Saudi Arabia; 7 Center for Research on Microgrids (UPC CROM), Department of Electronic Engineering, Barcelona East School of Engineering (EEBE), BarcelonaTech (UPC), 08019 Barcelona, Spain; 8 Catalan Institution for Research and Advanced Studies (ICREA), Pg. Lluís Companys 23, 08010 Barcelona, Spain; 9 Center for Research on Microgrids (AAU CROM), AAU Energy, Aalborg University, 9220 Aalborg East, Denmark

**Keywords:** Energy systems, Hybrid, Off-grid, Solar PV, Wind turbines, Hydrogen system, Sizing optimization, Deterministic approach, Electrical and electronic engineering, Energy infrastructure, Energy grids and networks, Hydrogen storage, Solar energy, Wind energy

## Abstract

The transition to sustainable power infrastructure necessitates integrating various renewable energy sources efficiently. Our study introduces the deterministic balanced method (DBM) for optimizing hybrid energy systems, with a particular focus on using hydrogen for energy balance. The DBM translates the sizing optimization problem into a deterministic one, significantly reducing the number of iterations compared to state-of-the-art methods. Comparative analysis with HOMER Pro demonstrates a strong alignment of results, with deviations limited to a 5% margin, confirming the precision of our method in sizing determinations. Utilizing solar and wind data, our research includes a case study of Cairo International Airport, applying the DBM to actual energy demands.

## Introduction

Hybrid off-grid systems, designed for longevity, possessed inherent complexities. Notably, integrating hydrogen as an energy storage solution amplified the challenges related to system sizing. While hydrogen offered remarkable energy density and could be produced from renewable sources, its high levelized cost of energy (LCOE) necessitated meticulous optimization to bring down the overall system LCOE. Batteries had been a predominant choice in hybrid systems, but the allure of hydrogen storage as a sustainable alternative was undeniable. Still, the harmonious interplay between wind and solar PV systems mitigated their energy production shortfalls, enhancing the system’s comprehensive reliability. Increasingly, these hybrid energy configurations were seen as the vanguard for sustainably powering remote and off-grid regions.

Research conducted in^1^ described the design information of solar PV and wind turbine hybrid power generation systems to provide electricity to a model community of 100 households and a health clinic and elementary school. The optimal simulation results in this study showed that solar PV/wind turbine/diesel generator/battery and converter was the best-configured system for their application with a renewable fraction of 84%. Many research papers have introduced different system configurations and comparative analysis for deciding the most economically feasible one. One of these researches in^2^ presented a case study in the desert region of the United Arab Emirates. This study introduced a technical-economic analysis based on integrated modeling, simulation, and optimization approach to design an off-grid hybrid solar PV/FC power system. This system was designed to meet the residential community’s energy demand of 4500 kWh/day (150 houses). The total power production from the distributed hybrid energy system was 52% from the solar PV and 48% from the FC with a 40.2% renewable fraction, which was a low value for the renewable energy penetration of this system. Consequently, one of the main concerns of our paper is how to achieve a renewable fraction of 100% in the simulated configurations of various hybrid off-grid systems. These given numbers of renewable fractions are used to provide a rough estimate for the previous research that focused on the penetration of renewable resources (increasing the renewable fraction percentage) and selecting the best configuration for the application targets.

Another approach for choosing the best size and location for off-grid hybrid systems was presented by^3^. They considered economic, technical, social, and environmental factors to discover the ideal capacity and location for continually meeting the load while reducing LCOE and overall life cycle cost. The hybrid algorithm based on the geographic information system, simulated annealing, and enhanced harmony search was evaluated with real data for a genuine case study in South Khorasan, Iran, and the findings showed that it provided more accurate results than those from previous heuristic approaches. Compared to a standalone diesel system, the hybrid system saved 8948 L of diesel generator fuel and reduced pollutant emissions by 59.6% according to the IHS SA-GIS methodology. Cai et al.^3^ and Alberizzi et al.^4^ presented a method based on mixed integer linear programming (MILP) and an algorithm implemented through Matlab software to determine the ideal size of a hybrid solar-wind system with battery storage to replace a diesel-fueled internal combustion engine (ICE) for a mountain lodge in South Tyrol, Italy. This gap in understanding the optimal sizing and location for hybrid systems, particularly in different geographical and environmental contexts, is a key focus of our study.

Research in^5^ conducted a reliability-based analysis of different combinations of photovoltaic panels and wind turbines with a backup system. The study aimed to compare the sizing of three hybrid energy systems: solar PV/Genset, Wind/Genset, and solar PV/Wind/Genset, focusing on reducing carbon dioxide emissions, total annual costs, or both. Utilizing the gravitational search algorithm, the study’s results were juxtaposed with those obtained from the simulated annealing method. This comprehensive examination encompassed 45 cases, integrating economic and environmental analysis and considerations of human health impacts due to carbon dioxide emissions. The findings revealed that the PV/Wind/Genset system was optimal, achieving a 27.2% reduction in pollution and a 4.76% cost saving compared to the solar PV/Genset system. Additionally, the imposition of carbon taxes on hybrid system designs proved effective, reducing CO2 emissions by about 9% and lessening health-related damages by 8.9%. While comprehensive, this study highlights the need to explore diverse optimization algorithms and system configurations further to enhance environmental and economic efficiencies in different geographical contexts.

Research work in^6^ introduced a novel method for optimizing power planning in renewable hybrid systems, including wind turbines, PV systems, bio-site units, thermal storage, and electric vehicle facilities with smart charging. Their approach minimized NPV and LCOE using a refined cost model and a modified multi-objective function. Bio-site units enhanced efficiency and reduced emissions, while adaptive smart charging reduced LCOE and NPV by optimizing load and storage requirements. The study also incorporated uncertainties in renewable sources, load demands, and electric vehicle aspects, adding robustness but increasing resource and storage needs, thus raising costs. They applied an advanced Grey Wolf Optimizer combined with the Sine Cosine Algorithm for better convergence and stability. The research highlights the ongoing challenge of balancing efficiency and cost-effectiveness in renewable energy systems, especially given inherent uncertainties, suggesting the need for more sophisticated optimization strategies.

**Figure 1:**
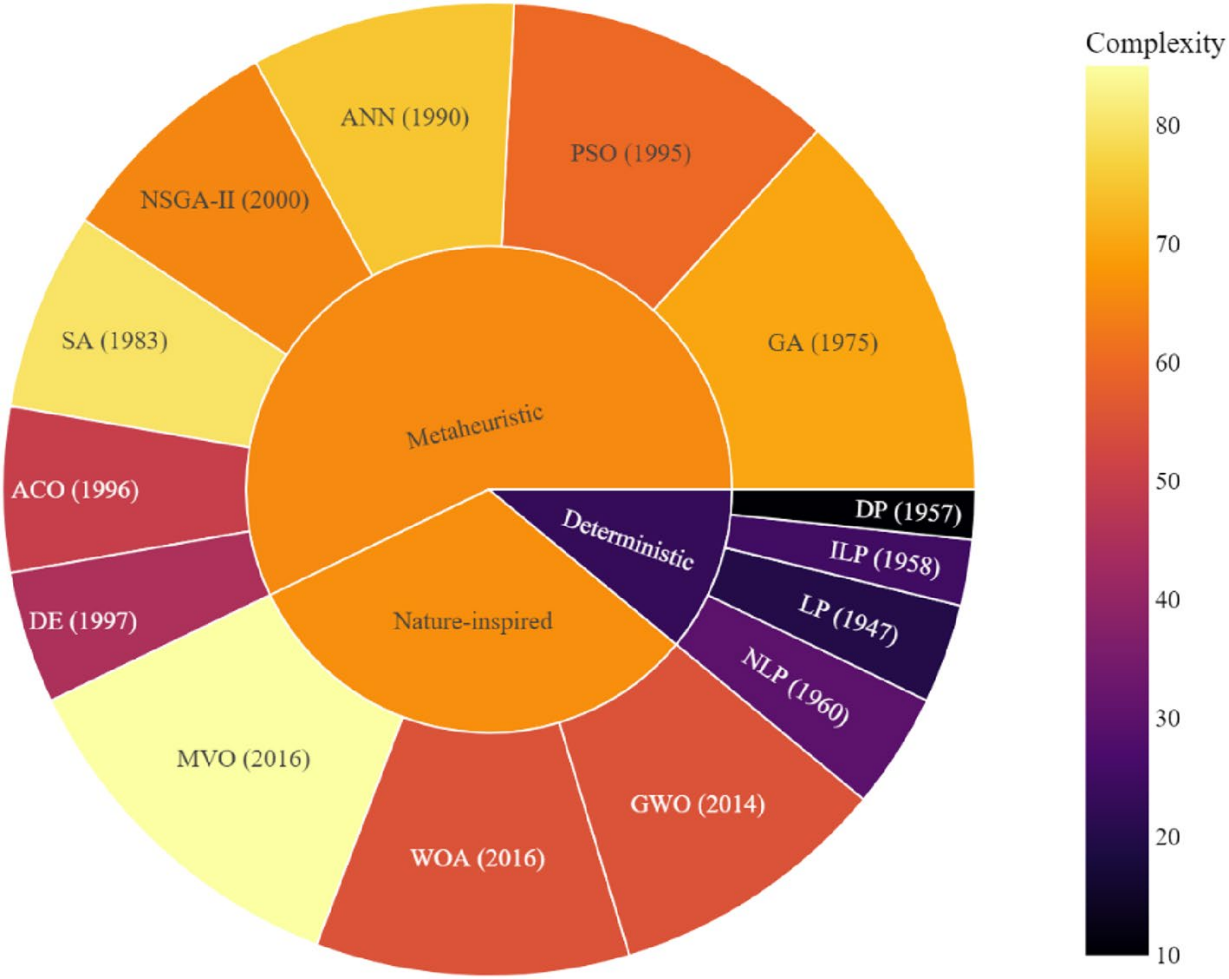
Methods used for solving the sizing optimization of energy systems. (Source: self painted by the author).

Figure 1 provides a visual taxonomy of optimization methods used in energy system sizing, classified by their nature and complexity. It illustrates a spectrum ranging from deterministic approaches like linear programming (LP), nonlinear programming (NLP), integer linear programming (ILP), and dynamic programming (DP), to nature-inspired and metaheuristic algorithms such as genetic algorithm (GA), particle swarm optimization (PSO), and ant colony optimization (ACO). The complexity gradient, denoted by the color bar, indicates that deterministic methods, while established earlier, tend to offer lower complexity relative to the more recent metaheuristic or nature-inspired methods. This suggests that deterministic methods, despite their simplicity, remain a robust choice for energy system optimization, especially in scenarios where computational efficiency and the guarantee of reaching an optimal solution are paramount.

**Figure 2:**
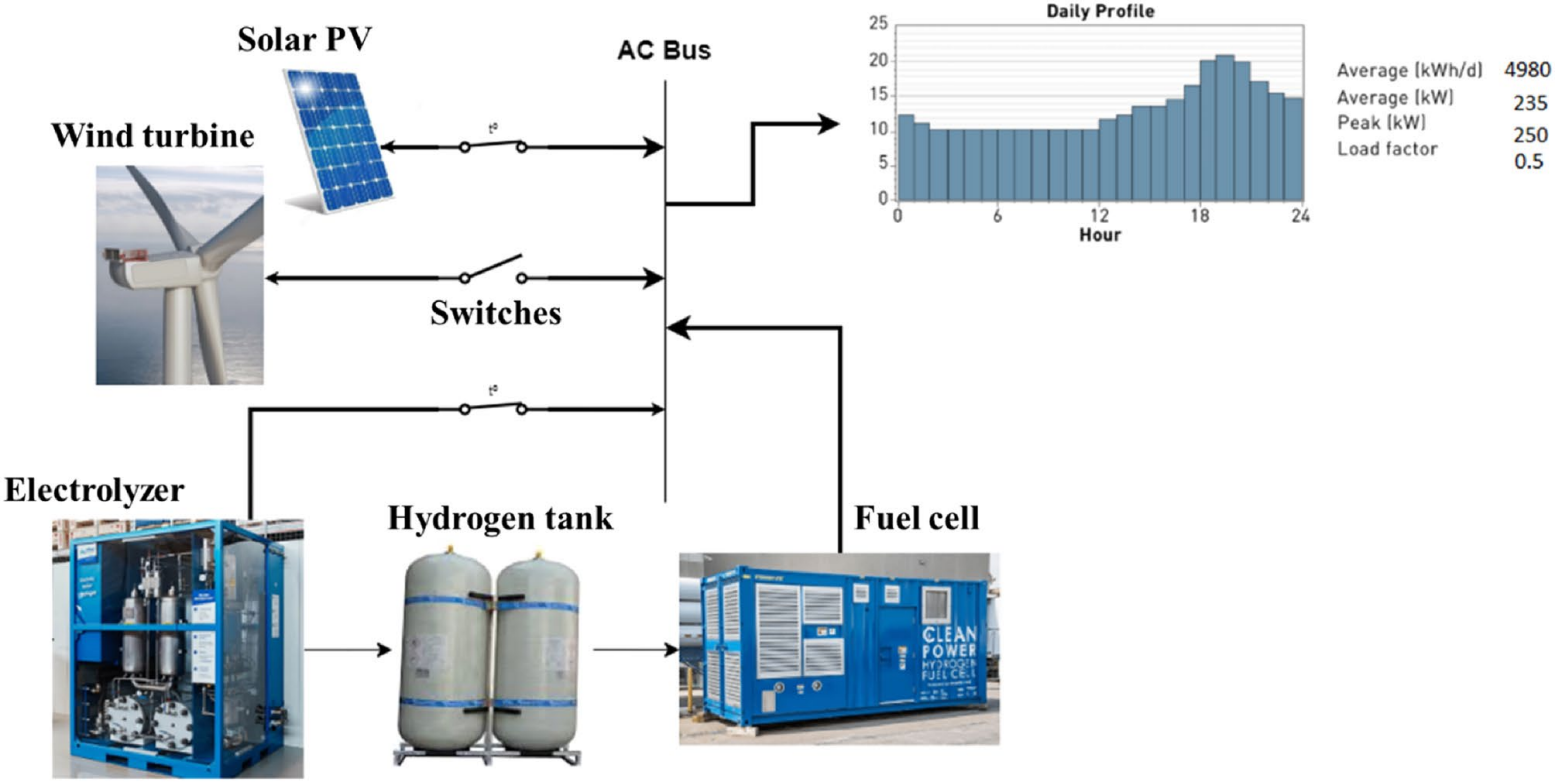
Conceptual model for energy system based on hydrogen storage.

Our study, as outlined in Table 1, addresses significant gaps in renewable energy system (RES) sizing optimization literature. It uniquely combines a deterministic methodology with hydrogen system sizing and computational efficiency, a combination not extensively explored in previous research. Our approach involves detailed 1-year simulations across diverse system configurations shown in Fig. 2, offering a comprehensive view of RES performance. A distinct feature of our research is the theoretical analysis of optimality, which, alongside thorough techno-economic assessments, provides deeper insights into RES optimization. Further, our findings are validated with commercial software.

**Table 1:** Comparison of research papers on RES sizing optimization.

Criteria	^7^	^8^	^9^	^10^	^11^	^12^	This Study
Deterministic methodology	$\checkmark$	$\checkmark$					$\checkmark$
Hydrogen system sizing				$\checkmark$		$\checkmark$	$\checkmark$
Computational efficiency					$\checkmark$		$\checkmark$
1-year Simulations	$\checkmark$	$\checkmark$	$\checkmark$	$\checkmark$		$\checkmark$	$\checkmark$
Configurations’ study	$\checkmark$		$\checkmark$		$\checkmark$	$\checkmark$	$\checkmark$
Theoretical analysis for optimality							$\checkmark$
Techno-economic analysis	$\checkmark$	$\checkmark$	$\checkmark$	$\checkmark$	$\checkmark$	$\checkmark$	$\checkmark$
Verification with commercial software	$\checkmark$		$\checkmark$	$\checkmark$	$\checkmark$	$\checkmark$	$\checkmark$

## System modeling

### Solar photovoltaic (PV) model

The main objective functions are used to get the output power of the PV modules taking into consideration the efficiency of the modules and other derating factors. The first equation used in PV modeling is an output power function of irradiance^13^.

1 $$\begin{aligned} P_{\text {pv}} = Y_{\text {pv}} f_{\text {pv}} \left( \frac{G_T(t)}{G_{T,\text {STC}}} \right) . \end{aligned}$$

Other factors that have been encountered during the literature review. Temperature and wind speed have significantly affected the model to get more accurate results feasible for execution. Consequently, another objective function for calculating the output power is evolved, providing a relation between the irradiance and temperature to deliver the actual output power shown in the equation^14^.

2 $$\begin{aligned} p = \eta T_{\text {ref}} A G_T \left[ 1 - 0.0045(T_c - 298.15)\right] . \end{aligned}$$

After studying the behavior of temperature and irradiance throughout the meteorological model, the effect of this behavior is linked to the changes in each of the two parameters and the overall efficiency of the PV system^14^. The following equation supports this relation.

3 $$\begin{aligned} \eta = \eta _{T_{\text {ref}}} \left[ 1 - B_{\text {ref}} (T_{c,i} - T_{\text {ref}}) \right] . \end{aligned}$$

Numerous factors prohibit solar PV arrays from operating at maximum efficiency. In addition to voltage drop and dust accumulation, one of these factors is the operating temperature of the solar PV module, which can contribute considerably to the most significant proportion of power loss. The effect of temperature on output varies by module and can be calculated using the temperature coefficients supplied on the manufacturer’s data sheets and the following relationships.

4 $$\begin{aligned} T_m&= 20.4 + 1.2 \times T_a , \end{aligned}$$

5 $$\begin{aligned} T_x&= 1 + \alpha (T_m - T_a) . \end{aligned}$$

In this model, it is assumed that AC losses are fixed at roughly 7% while array temperature losses range between 5 and 11% depending on the monthly temperature profile for theexample shown in Table 2.

**Table 2:** Monthly variations in temperatures associated losses in Cairo-2023.

Month	$T_a$	$T_m$	$T_x$	Losses (%)
Jan.	14.43	37.71	0.96	4.21
Feb.	15.44	38.93	0.95	4.61
Mar.	18.55	42.65	0.94	5.84
Apr.	21.51	46.21	0.93	7.02
May	25.41	50.90	0.91	8.57
Jun.	27.85	53.82	0.90	9.54
Jul.	29.46	55.75	0.90	10.18
Aug.	29.28	55.53	0.90	10.11
Sep.	27.25	53.10	0.91	9.30
Oct.	24.34	49.61	0.92	8.14
Nov.	19.64	43.97	0.94	6.28
Dec.	16.12	39.75	0.95	4.88

### Wind turbine generators

**Figure 3:**
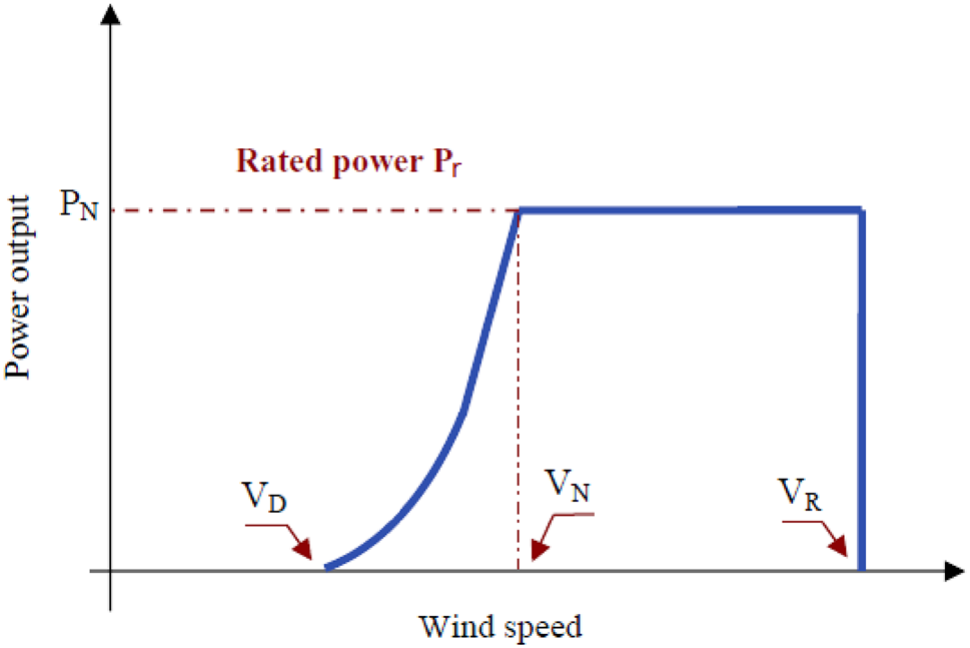
Power output curve of wind^15^.

The wind turbine generator (WTG) hourly power output, at the studied location, depends on the hourly wind speed as shown in Fig. 3. It can be expressed by following equations^15^.

6 $$\begin{aligned} P_{\text {W}}(V_V) = {\left\{ \begin{array}{ll} P_r (A + B V_V + C V_V^2) &{} \text {if } V_D \le V_V \le V_N \\ P_r &{} \text {if } V_N \le V_V \le V_R \\ 0 &{} \text {elsewhere}. \end{array}\right. } \end{aligned}$$

7 $$\begin{aligned} A&= \frac{1}{V_D^2-V_R^2}\left[ V_D\left( V_D+V_R\right) -4\left( V_D V_R\right) \left( \frac{V_D+V_R}{2 V_R}\right) ^3\right] , \end{aligned}$$

8 $$\begin{aligned} B&=\frac{1}{V_D^2-V_N^2}\left[ 4\left( V_D+V_R\right) \left( \frac{V_D+V_N}{2 V_N}\right) ^3-3\left( V_D+V_N\right) \right] , \end{aligned}$$

9 $$\begin{aligned} C&= \frac{1}{V_D^2-V_N^2}\left[ 2-4\left( \frac{V_D+V_N}{2 V_N}\right) ^3\right] , \end{aligned}$$

10 $$\begin{aligned} P_{W T}(t)&= N_W \cdot P_W(t). \end{aligned}$$

We analyze the site’s wind conditions and match them with the desired turbine characteristics to select the appropriate wind turbine model for a specific location. Using computational tools, we determine the optimal rated speed and the cut-in and cut-out speed limits. With these parameters defined, we consult a comprehensive database of available wind turbines^16^ to find a model that aligns with our requirements. We further refine our selection based on site-specific restrictions, such as height limitations.

### Hydrogen system

FC can be defined as an electrochemical device that produces electrical power directly from a fuel like hydrogen, natural gas, diesel, or propane. Its operation is similar to that of a conventional battery except for some parts that will be discussed in detail later in this paper and will affect the modeling of the hybrid off-grid system. Accordingly, their development has been much related to the development of electrochemistry more than power engineering, and it is already studied as a distinct branch of physical chemistry^17^. The second element in the hydrogen system is the ELZ, an electrochemical device that makes electrolysis for the water molecules to produce hydrogen and oxygen. The excess electrical in the system powers this process. In other words, ELZ is used to convert unused electrical energy into stored chemical energy inside hydrogen and then recall it back in the time of operation. The mathematical formula of the produced hydrogen can be expressed as follows^18^.

11 $$\begin{aligned} E_{H_2}= & {} \eta _{\text {Elz}} \times \left( \frac{HV_{H_2}}{\rho _{H_2}} \right) , \end{aligned}$$

12 $$\begin{aligned} M_{H_2}= & {} \frac{P_{\text {Elz}}}{E_{H_2}}. \end{aligned}$$

The sizing of the hydrogen storage system takes place after determining the maximum energy generation from the PV, WTGs, and the minimum load power. The ELZ utilizes surplus energy to produce a maximum of 23 kg of hydrogen per hour. Consequently, the hydrogen tank size is determined to have a maximum capacity of 100 kg, representing the upper safe limit the hydrogen might reach during the year. To decrease the volume of a gas at constant temperatures, one can increase its pressure. Under such high pressure, a 125-l tank can store 5 kg of hydrogen. Currently, most car manufacturers opt to store hydrogen in gaseous form at high pressure. This technology allows a FC-powered car to cover between 500 and 600 km between fill-ups, as referenced in^19,20^. The Model H15T4X200^21^ compresses hydrogen in each 10 kg tank to 345 bar. The system configuration includes ten compressors paired with ten hydrogen tanks, plus an additional tank for excess hydrogen storage. As a result, the total hydrogen storage capacity stands at 100 kg, maintained at a constant pressure of 345 bar. This system connects to 10 compressors via pipelines, ensuring the pressure for hydrogen storage and transport.

### Load profile system

**Figure 4:**
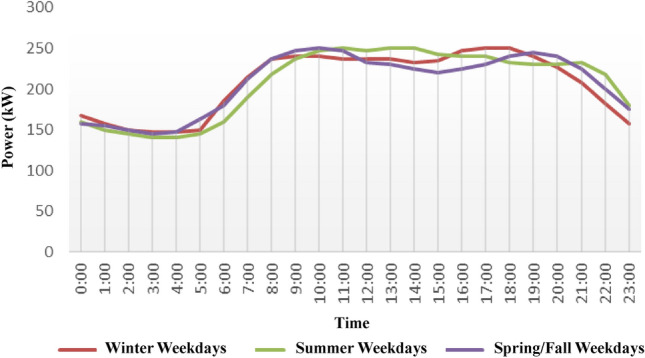
Load profile system based on RTS system.

In this study, it is assumed that the DC and AC wiring losses are small enough to be neglected due to the small geographic scatter of the study system. The reliability test system (RTS) load profile is used in this study^22^ as shown in Fig. 4, and the peak load is set to 250 kW. The load profile is then determined per hour for the 8760 h of a year.

### Economic investigation for the studied system

The economic evaluation of hybrid renewable energy systems is pivotal, specifically assessing LCOE, capital costs, and cash flows to determine project viability. LCOE represents the unit cost of electricity over the system’s lifespan, while the net present cost (NPC) reflects the discounted total of all expenses incurred throughout the project’s life. These financial metrics are critical for investors considering funding such ventures, especially in remote areas.

A detailed cash flow analysis elucidates operational, maintenance, and, where applicable, fuel costs. The discount factor, distinct from inflation, adjusts future costs to present-day values, with the real discount factor accounting for inflationary effects. The capital recovery factor (CRF) translates NPC into an equivalent annual cost influenced by inflation and project duration. These economic indicators form the foundation for optimizing techniques to minimize costs and achieve the most economical system configuration^23^.

To optimally design the hybrid generation system, the optimization problem, defined by equation below as in^23^, is solved using any of the mentioned optimization techniques in the paper.

13 $$\begin{aligned} C_{\text {cpt}}= & {} N_{\text {pv}} \times C_{\text {PV}} + N_{\text {Tank}} \times C_{\text {Tank}} + C_{\text {FC/ELZ}} + N_{\text {conv}} \times C_{\text {conv}}, \end{aligned}$$

14 $$\begin{aligned} C_{\text {Mtn}}= & {} \frac{(i+1)^n - 1}{i(1+i)^n} \left( N_{\text {PV}} \times C_{\text {PV,Mtn}} + C_{\text {FC,Mtn}} + C_{\text {Elz,Mtn}} \right) , \end{aligned}$$

To minimize the total cost function,

15 $$\begin{aligned} C_T = C_{\text {cpt}} + C_{\text {Mtn}}. \end{aligned}$$

The economic model of the studied system is studied for the lifetime of the system using the following equations:

16 $$\begin{aligned} O{ \& }M \text { costs}({\$}/\text {year})&= AEP \times O{ \& }M \text { costs}, \end{aligned}$$

17 $$\begin{aligned} AR&= AEP \times ESP \quad ANI = AR - O{ \& }M \text { costs}({\$}/\text {year}), \end{aligned}$$

18 $$\begin{aligned} NPV&= \sum _{k=1}^{n=20} \frac{ANI}{(1+R)} - CI, \end{aligned}$$

19 $$\begin{aligned} PVM&= \sum _{k=1}^{n=20} \frac{NPV}{(1+R)} - CI , \end{aligned}$$

20 $$\begin{aligned} PV^*&= \frac{FV}{(1+R)^P}, \end{aligned}$$

21 $$\begin{aligned} LAC&= \frac{-PVC \times R}{1 - (1+R)^{-P}} , \end{aligned}$$

22 $$\begin{aligned} PVC&= PVDC + PVM + CI, \end{aligned}$$

23 $$\begin{aligned} LCOE&= \frac{LAC}{AEP} . \end{aligned}$$

## Deterministic balance method (DBM)

**Figure 5:**
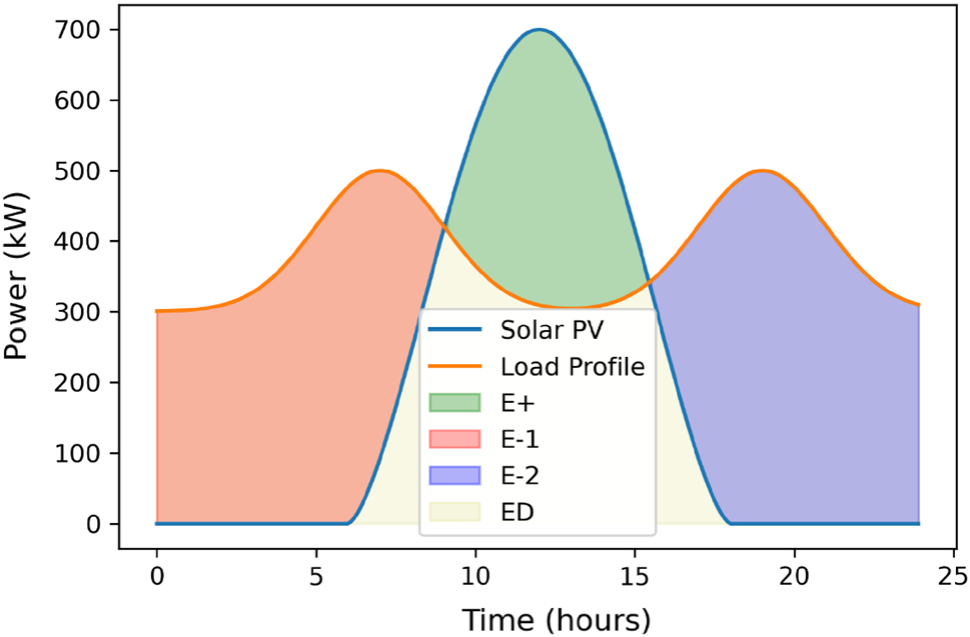
Area under curve for DBM.

### Methodology procedures

**Figure 6:**
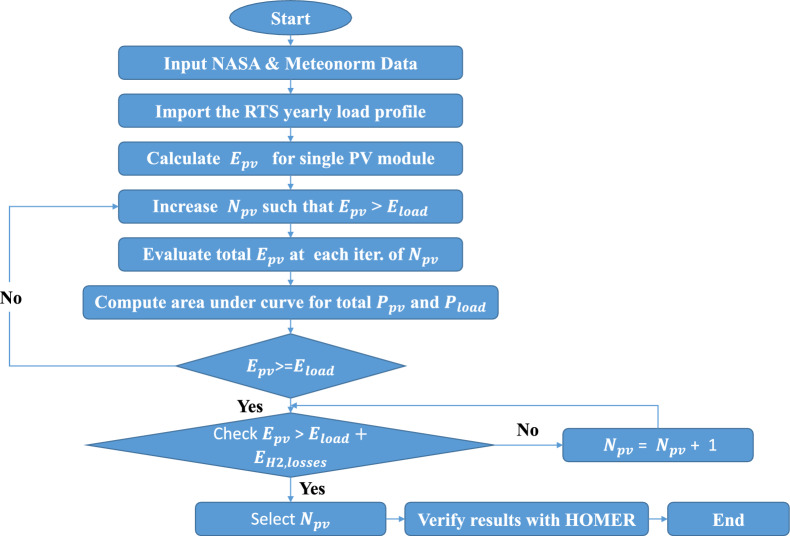
Low chart of the DBM method.

The use of DBM requires determining solar PV output power, ELZ size, FC size and hydrogen tank efficiency models. The accuracy of the solar PV model is crucial since it will be used to guide the overall layout of the system. To use the DBM, it is necessary to balance the energy need to offset the time of zero solar PV output hour (usually at night) with the energy produced net by the solar PV (during sun hours after being absorbed through the load). Hydrogen tanks will store this surplus energy until needed; at this point, FCs will convert it to meet the day’s energy needs. Power generated by solar PV and required by the load through FCs is determined using the Area under graphs, as illustrated in Fig. 5, which are computed using the trapezium rule. A discrete optimization is performed to determine how many solar PV modules would be ideal for striking this equilibrium. In the first stage of the DBM design process, N solar PV modules would be sufficient to meet the required load and provide excess power at the end of the day. To begin computing energy Production values, this is founded on a random guess. This initial estimate is based on the energy balance equation discussed in the prior work^24^ and is performed to estimate the surplus power supplied by solar PV to compensate for the time of zero PV output power completely. Using the data from NASA and METEONORM^25,26^, the output solar PV power is calculated using equations in the section of modeling of solar PV based on the irradiance profile of a specific day. Throughout the entirety of the model, the method of calculating energy production by obtaining the Area under curves is the primary method employed. Numerous methods for calculating the Area under curves, including the trapezium rule and the integration of curve functions in MATLAB. This model uses the trapezium rule to calculate the area under curves in a 1-h time step because it is challenging to obtain the function of each curve so that it can be integrated. In addition, the accuracy of the trapezium rule is deemed acceptable with minimal error ranges. As shown in Fig. 6, the Area under the curve is calculated for the $E^+$, $E^-$, and $E^D$.

24 $$\begin{aligned} E^{-,1}&= \int (P_l - P_{pv}) \, dt,&\text {for } t_{s,1}< t< t_{e,1}, \text { where } P_{pv} < P_l \end{aligned}$$

25 $$\begin{aligned} E^{-,2}&= \int (P_l - P_{pv}) \, dt,&\text {for } t_{s,2}< t< t_{e,2}, \text { where } P_{pv} < P_l \end{aligned}$$

26 $$\begin{aligned} E^{+}&= \int (P_{pv} - P_l) \, dt,&\text {for } t_{s,+}< t< t_{e,+}, \text { where } P_l < P_{pv} \end{aligned}$$

27 $$\begin{aligned} E^{D}&= \int P_l \, dt,&\text {for } 0< t< t_{e}, \text { where } P_l < P_{pv} \end{aligned}$$

$E^{+}$ has to be equal or greater than $E^{-}$ to ensure the energy balanced operation.

Our target is to find the optimal sizing of $N_{PV}$ as follows:

28 $$\begin{aligned} N_{PV} = \frac{E^{+} + E^D}{E_{1,PV}}. \end{aligned}$$

**Figure 7:**
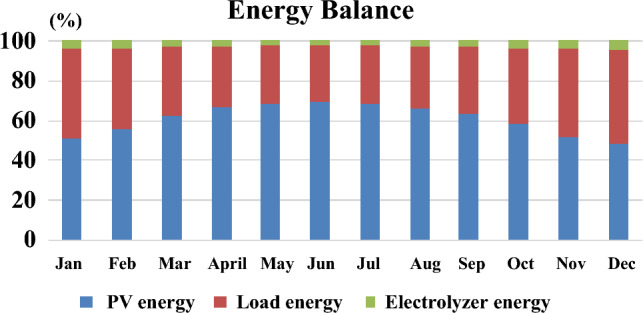
Energy balance results using DBM.

Figure 7 depicts the computation of the optimal number of solar PVs. For Eq. (28), we iteratively compute the number of PVs, denoted as $N_{PV}$, given the energy provided by each PV module, $E_1$. This calculation is grounded on the premise that these values represent the maximum energy required by the load during nighttime hours. The goal is to achieve an optimal surplus of solar PV power capable of covering the load, taking into account the efficiencies of the FC and the hydrogen tank.

Furthermore, it is observed that the surplus energy supplied by the solar PV modules must surpass the nighttime discharge power by a specific margin. This surplus predominantly hinges on the efficiency of the fuel cell, electrolyzer, and hydrogen tank. As such, system components with higher efficiency tend to yield better sizing optimization results. Equation (29) elucidates the influence of system component efficiencies on the surplus energy supplied and serves as a constraint for the optimization strategy.

29 $$\begin{aligned} E^-&= E^+ \cdot \eta _{FC} \cdot \eta _{H_{2},Tk}. \end{aligned}$$

$E^{D,1}$ and $E^{D,2}$ resemble the energy the load consumes via solar PV modules during the first hours between sunset and sunrise. In previous calculations, these two values should have been taken into account. In our system, $E^{-}$ represents the energy available to the ELZ. The hydrogen production, $H_2$, is governed by Eqs. (11) and (12), derived from the energy supplied by $E^{-}$. Subsequently, the produced $H_2$ serves as the primary input for the FC. The sizing or rating of the FC is directly determined based on the volume of hydrogen generated by the ELZ. Nevertheless, it is essential to analyze the system efficiency, as they will account for the losses that occur during the conversion process via hydrogen tanks and FCs. After determining the optimal sizing of the system components, we proceed to calculate the annual energy production. This calculation is grounded in the operational characteristics of each component, as described by the subsequent equations. It is essential to note that the performance of each component directly influences the overall system’s energy output. By integrating these operational equations over the year, we obtain an accurate representation of the system’s annual energy production capabilities.

It is essential to note that, despite the method designation, the DBM does not prioritize addressing uncertainties. Instead, it is engineered primarily to tackle the sizing dilemma of hybrid energy systems deterministically. Interestingly, DBM focuses on determining the optimal size analytically, operating much in the vein of MILP solvers. However, its augmented flexibility sets it apart, allowing users to adjust sizing parameters based on seasonal variations they input. One of its distinctive advantages is its capability to reduce the iteration counts typically required and deliver a feasible solution. Such a solution could be computationally burdensome when sought through other heuristic methods. This section presents the methodology for calculating the optimal number of solar PV modules. Using this framework, one can similarly ascertain $N_W$ the ideal number of wind turbines. By employing data analysis and performance metrics, this approach ensures optimal energy output and system efficiency.

### Global optimality and computational complexity analysis

The objective function $N_{PV}$ is a linear function of $E^+$ and $E^D$, as $E_{1,PV}$ is constant. A linear function is convex. To establish this, consider two arbitrary values $E^{+,1}, E^{+,2}$ and $E^{D,1}, E^{D,2}$ representing the energy values on two different times of the day, and any $\lambda$ such that $0 \le \lambda \le 1$. The objective function must satisfy:

30 $$\begin{aligned} N_{PV}(\lambda E^{+,1} + (1 - \lambda )E^{+,2}, \lambda E^{D,1} + (1 - \lambda )E^{D,2}) \le \lambda N_{PV}(E^{+,1}, E^{D,1}) + (1 - \lambda ) N_{PV}(E^{+,2}, E^{D,2}) \end{aligned}$$

Given $E_{1,PV}$ is constant, we can simplify the above to:

31 $$\begin{aligned} \frac{\lambda E^{+,1} + (1 - \lambda )E^{+,2} + \lambda E^{D,1} + (1 - \lambda )E^{D,2}}{E_{1,PV}} = \lambda \frac{E^{+,1} + E^{D,1}}{E_{1,PV}} + (1 - \lambda ) \frac{E^{+,2} + E^{D,2}}{E_{1,PV}} \end{aligned}$$

This confirms the convexity of the objective function. The constraints involve integrals of the difference between measured values $P_l$ and $P_{pv}$, which form a set of linear equations. Linear equations define a convex set. The energy balance constraints can be expressed as linear inequalities, which also define convex sets. Therefore, the optimization problem, which aims to determine $N_{PV}$ such that the energy produced matches or exceeds the energy demanded, is convex. Since the objective function is convex and the feasible region defined by the constraints is a convex set, any local minimum found in this optimization problem is also a global minimum. The non-triviality of this optimization problem arises from the computational complexity involved in integrating measured values of power to obtain $E^+$ and $E^D$ for each hour across an entire year. The integrals do not have closed-form solutions and must be numerically evaluated. The computational complexity of the DBM algorithm is given by $O(m)$, with $m$ being the number of iterations until the energy balance is achieved. The algorithm’s steps and their complexities are as follows:

1. Calculate the energy output for a single PV module, $E_{1,PV}$, which is a constant. This is an $O(1)$ operation.
2. In each iteration $i$, the cumulative energy $E^+_i$ and the demand $E^D_i$ are computed. These are based on pre-determined measurements, the complexity is $O(1)$ per iteration.
3. The iteration proceeds until the condition $E^+_i \ge E_{Load} + E_{sys,losses}$ is met. Thus, the loop runs $m$ times, where $m$ is the smallest integer satisfying the energy balance.
4. The integral computation within each iteration has a polynomial complexity with respect to the number of data points; however, since it is a single operation per iteration, it does not affect the overall linear complexity.

The overall complexity is modeled by the equation:

32 $$\begin{aligned} O_{total} = O(1) + m \cdot O(1) = O(m) \end{aligned}$$

This equation demonstrates that the primary factor determining complexity is the number of iterations $m$, leading to a linear computational complexity of $O(m)$. The algorithm is efficient as it avoids unnecessary computations beyond the point of achieving an energy balance, ensuring minimal computational burden.

## Numerical results

### Sizing methodology of hybrid solar PV/hydrogen system: case study 1

#### Applying DBM for the system sizing

Our previous research derived the optimal sizing for the specified system using DBM. The finalized system comprised a total of $N_{\text {PV}} = 5400$ panels. Alongside this, the system configuration included FC with a capacity of 300 kW, ELZ with capacities of 800 kW and 1000 kW, respectively, and a hydrogen ($H_{2}$) tank with a storage limit of 100 kg. The efficacy and specifications of this model were compared with the system advisor model (SAM). For a detailed understanding of the model and its derivations, readers are referred to our earlier publication^24^.

**Figure 8:**
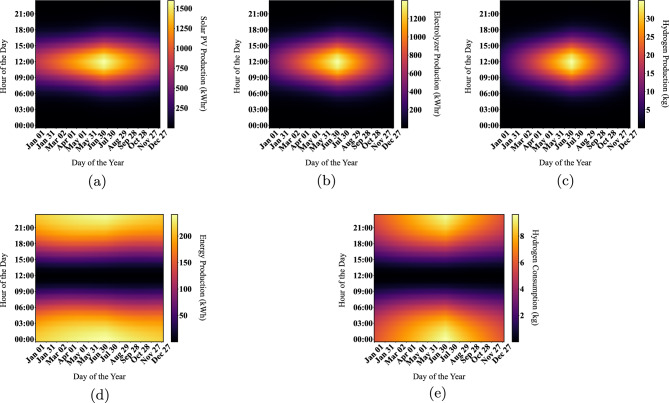
Visualization of system energy outputs using DBM.

In analyzing the annual system behavior for the examined location of Cairo International Airport, the production and energy consumption are investigated (same data as in^24^) using the DBM method. From October to March, hydrogen consumption exceeds production, and solar energy is the sole source of hydrogen production. During April through September, solar energy increases due to increased irradiance and moderate temperature ranges, causing hydrogen production to exceed consumption. The entire year’s excess solar electricity is stored in a hydrogen tank, yielding approximately 2000 kg of hydrogen that will be exported to the grid upon application of the power management method during January and December, when there is no surplus power and end consumers consume all produced energy. As shown in Fig. 7 and Table 3, the net energy produced by solar PV modules (considering temperature losses that are mentioned in previous section) is sufficient to cover the load demand for each month except for December that is considered as the worst-case scenario. The solar PV contribution in that energy mix is found to be 50% or exceeding depending on the month of the year and this what proves the capability of the DBM for optimizing the generation and meeting the load requirement. In December, there is a lack of generation around 5% which are supplied by the stored hydrogen from the excess of the previous months. Finally, energy profiles for solar PVs, FC, ELZ and hydrogen production are deduced using DBM for 8760 h as shown in Fig. 8.

**Table 3:** solar PV array temperature losses.

Month	Energy (kWh)	Losses (%)	Net energy (kWh)
Jan.	147,322.66	11.2	130,811.72
Feb.	168,392.6	11.61	148,842.41
Mar.	242,436.58	12.84	211,299.15
Apr.	285,159.56	14.02	245,176.85
May	323,613.87	15.57	273,222.64
Jun.	334,147.5	16.54	278,885.87
Jul.	329,821.4	17.18	273,164.21
Aug.	302,216.1	17.11	250,517.83
Sep.	257,057.1	16.3	215,150.56
Oct.	208,144.1	15.14	176,621.23
Nov.	153,297.2	13.28	132,939.74
Dec.	134,767.4	11.88	118,755.30

**Figure 9:**
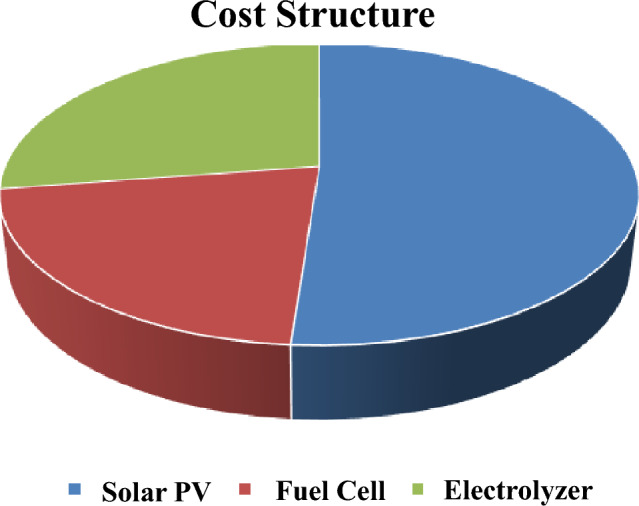
Cost structure of hybrid solar PV/hydrogen system).

Referring to cost equations in the modeling section and system cost per 1 kW as illustrated in Table 5, the economic model of the hybrid system is computed as shown in Table 4 and Fig. 9. Consider Electrical selling price (ESP) = 0.0788 USD/kWh^27,28^, project lifetime = 25 years, annual discount rate = 10% and assuming no decommissioning cost paid. Net present cost is negative, indicating that the project will not be profitable with the current selling prices for Solar PV plants announced by the government. The tariff prices will drastically increase if the hydrogen system is used as an energy storage system.

**Table 4:** Cost analysis of the system.

Month	Solar PV	Fuel cell	Electrolyzer	Hydrogen tank
Capital cost ($)	1,793,000	750,000	960,000	2000
Maintenance cost ($)	–	816,934	2,178,490	–
Replacement cost ($)	–	750,000	960,000	–
AR ($)	228,928			
ANI ($)	109,111			
NPV ($)	− 2,514,595*			
PVM ($)	2,995,424			
PVC ($)	6,500,424			
LAC ($)	716,139			
LCOE ($/kWh)	0.247			

#### Evaluation using HOMER tool

**Figure 10:**
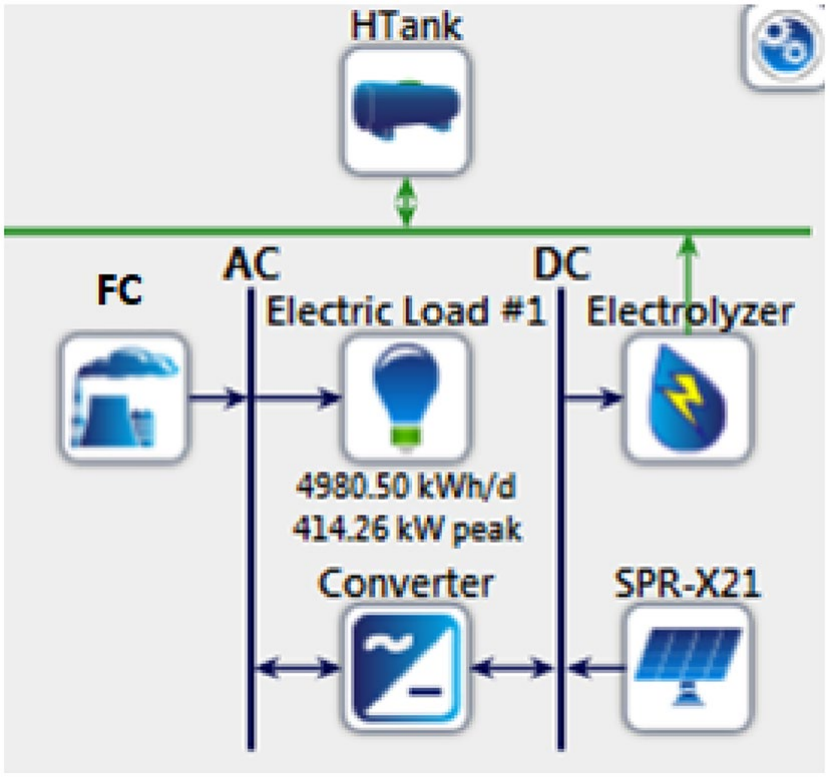
HOMER configuration (surplus mode).

HOMER-Pro^29^ is an advanced optimization model that conducts numerous simulations to determine the optimal system design. The PV/FC configuration, as depicted in Fig. 10, is simulated for Cairo International Airport using a yearly load profile provided in^24^. The simulation is executed on an HP laptop with an Intel®Core^TM^ i5 CPU at 2.20 GHz and 4.00 GB of RAM. Detailed specifications of the hybrid system components are illustrated in Table 6, where data has been collected from commercial datasheets during the selection process.

**Table 5:** System components specifications.

Parameter	Photovoltaic modules	Hydrogen tank
Rated power (kW)	0.335	Capacity: 100 kg
Type	monocrystalline	Efficiency: 98%
Abbreviation	SPR-X21	Lifetime: 15 years
Panel type	Flat type	Initial level: 10% relative to size
Rated capacity (kW)	0.335	Capital: $14/kg
Temperature coefficient	− 0.3	Replacement: $14/kg
Operating temperature (C)	43	O &M: $10/year/kg
Efficiency (%)	21	
Manufacturer	Sun power	
Model	SunPower X21-335-BLK	

Parameter	Fuel cell	Electrolyzer
Nominal power	250 kW	300 kW
$H_{2}$ consumption/production rate	5800 Btu/kWh	$H_{2}$ 60 Nm^3^/h
Input/output pressure	15 psig	10–27 barg
AC power consumption/production	5	5 kWh/Nm^3^
Nominal efficiency	90%	80%
Manufacturer	ES5-EA2AAN	Hydrogenics
Model	Bloom energy	HySTAT-60-10
Capital	$3000/kW	$1200/kW
Replacement	$3000/kW	$1200/kW
O &M	$0.01/h/kW	$100/year/kW

An optimization analysis determines the most efficient hybrid power system configuration that satisfies the set constraints at the minimal net present cost using the cycle charging control (CC). HOMER has three primary controllers: cycle charging, combined dispatch, and load following. Cycle charging is the most suitable for this application because it optimally employs the hydrogen system. In contrast, the other controllers do not incorporate the ELZ and hydrogen tank in their simulations. The hybrid power system derives its electrical production primarily from the solar PV system, contributing 76.2%, and the Genset (fuel cell), providing 23.8%, or 748,235 kWh/year. This represents a 100% renewable fraction. The hybrid system caters to the AC primary load, which is 1,582,615 kWh/year (58% of the total production). Additionally, the ELZ consumes 1,063,082 kWh/year (42%) from the hybrid system for hydrogen production, with a 214 kWh/year surplus. Consequently, the unmet electrical demand by Solar PV—the primary power source—stands at 235,003 kWh/year. The system also generates an excess electricity of 366,370 kWh/year. This data plays a pivotal role in determining the hybrid system’s reliability.

**Figure 11:**
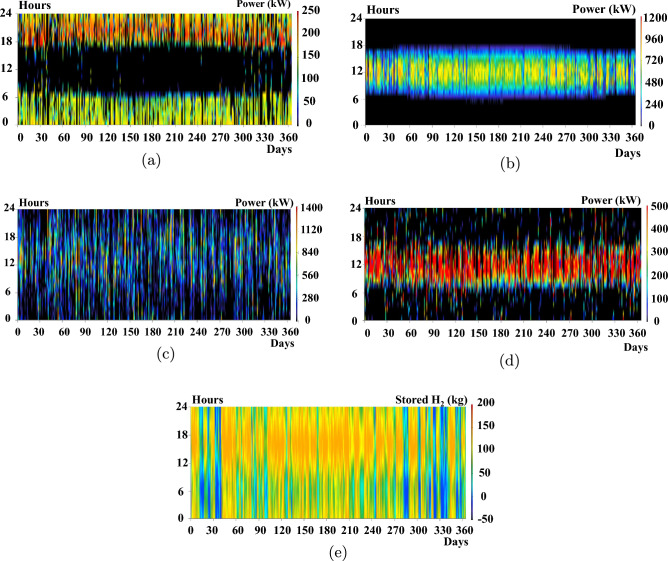
System operation profiles for 1-year simulations using HOMER.

To corroborate these results, HOMER Pro creates energy profiles for the solar PV, FC, ELZ, and hydrogen tanks, as depicted in Fig. 11. The operational ranges are 0–1800 kW for Solar PV, 0–250 kW for the FC, and 0–700 kW for the ELZ. These findings align with the operational ranges derived from the DBM method, as illustrated in Fig. 8. However, a deviation appears in the hydrogen tank storage, which fluctuates between 5 0 and 200 kg, differing from the DBM results. This discrepancy arises from the lack of an effective power management controller as in^30^ that streamlines hydrogen production while avoiding undue stress on the hydrogen tanks, as evident in Fig. 11(e) with a significant portion of the regions appearing red during the summer.

### Sizing methodology of solar PV/wind/hydrogen system: case study 2

#### Applying DBM for the system sizing

**Figure 12:**
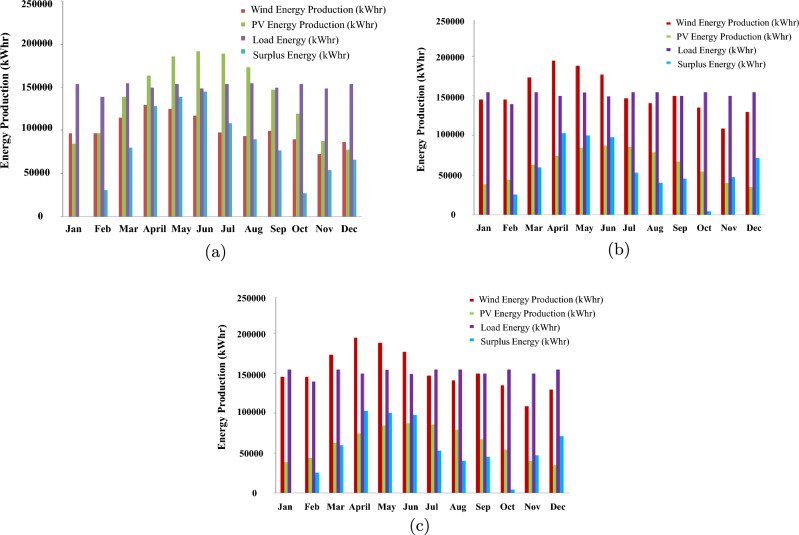
Comparative analysis of energy production scenarios.

This study analyzes various hybrid renewable energy scenarios, focusing on different combinations of WTGs and solar PV systems. We start with a 250 kW WTG and increase its capacity in 250 kW increments to 750 kW. Simultaneously, we assess each WTG level’s required solar PV contribution to ensure balanced power generation. Our analysis identifies January as a critical month due to its lack of surplus energy. Using this information, we optimize system sizing and energy output predictions for January using the DBM approach. The results in Fig. 12 illustrate the various configurations between solar PV and WTGs.

**Figure 13:**
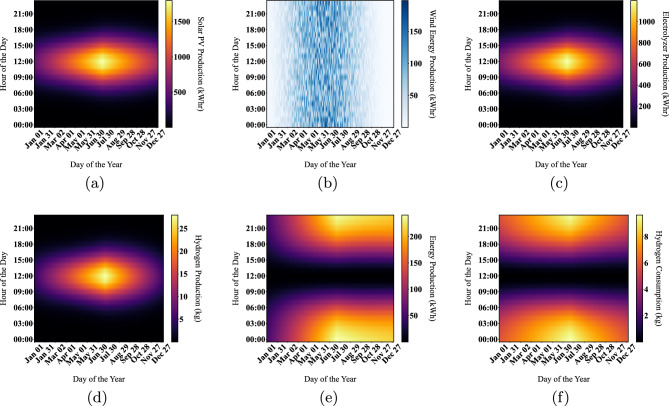
Comparison of various energy outputs using DBM.

System in Fig. 12c is found to have the highest annual energy production with a higher ratio of solar PV installed capacity compared to the installed wind turbine. As a result, this system is used in this paper for full simulation for 8760 h using DBM and conducting energy profiles for all system components, as shown in Fig. 13.

#### Sensitivity analysis

**Figure 14:**
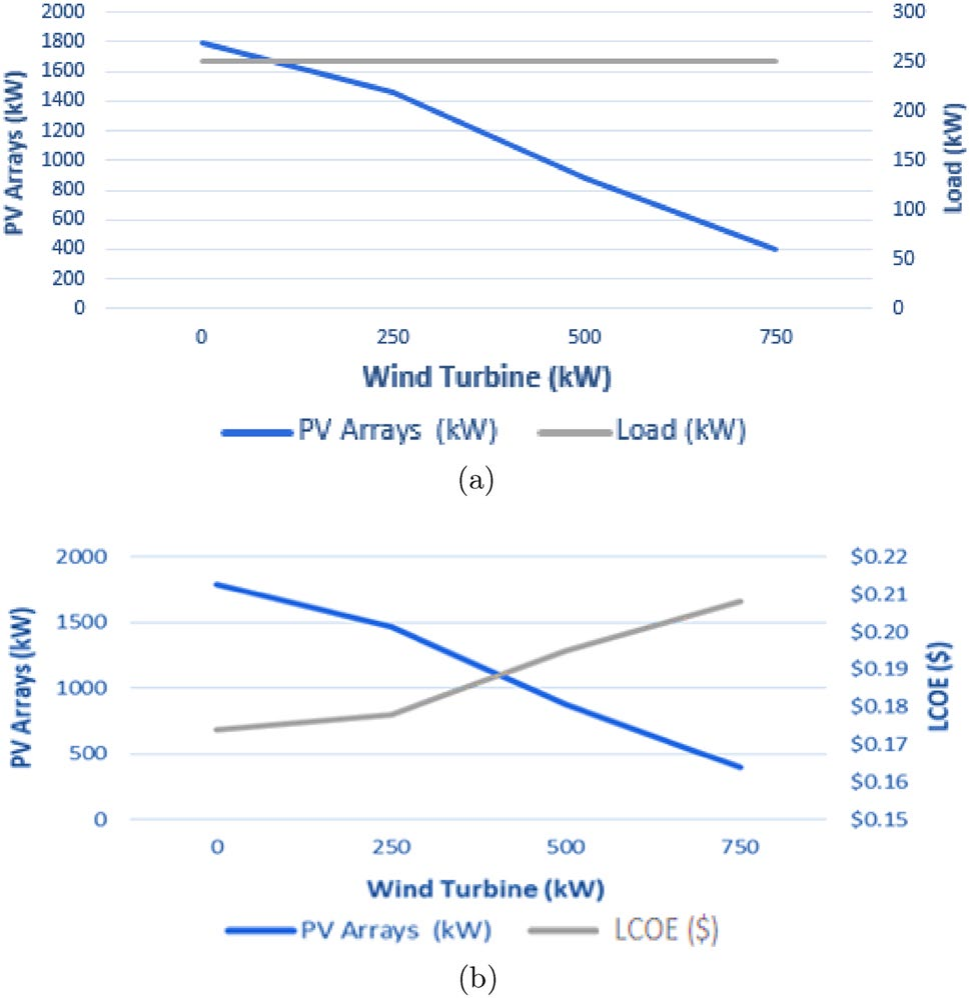
Comparative analysis of solar PV and WTGs.

Sensitivity analysis plays a pivotal role in understanding the intricacies of system design, specifically in determining how the ideal composition of components shifts in response to parametric fluctuations over a system’s lifespan. Figure 14 presents surface plots that depict variations in solar PV array capacity and wind turbine quantity related to primary load adjustments. For a more narrowed focus, we consider two specific sensitivity cases: the installed capacities of solar PV and wind turbines. Meanwhile, we maintained the other two variables-load and price-at a constant, pegging them at 100%, and analyze the outcomes within the context of the maximum renewable fraction.

**Figure 15:**
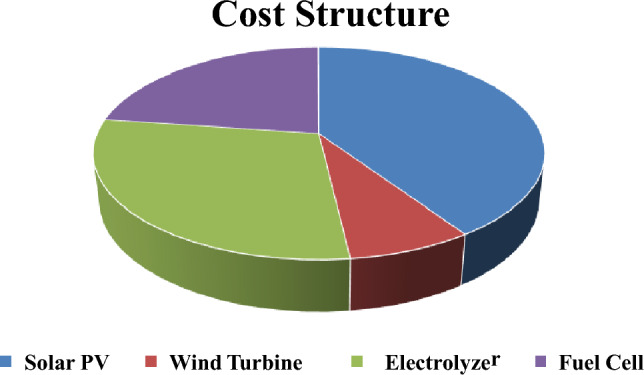
Cost structure of hybrid solar PV/WTG-system 1.

Consider ESP = 0.0788 USD/kWh^27,28^, project lifetime = 25 years, annual discount rate = 10% and assuming no decommissioning cost paid. Additionally, a sensitivity analysis is conducted as shown in Fig. 15 and Table 6 for studying the effect of ratio between solar PV and wind installed capacity on the LCOE.

**Table 6:** Cost analysis of the system.

Month	Solar PV	Wind turbine	Electrolyzer	Fuel cell	Hydrogen tank
Capital cost ($)	1,340,000	250,000	960,000	750,000	2,000
Maintenance cost ($)	–	113,463	2,178,490	816,934	–
Replacement cost ($)	–	–	960,000	750,000	–
AR ($)	263,678				
ANI ($)	139,323				
NPV ($)	− 2,037,360				
PVM ($)	3,108,886				
PVC ($)	6,410,886				
LAC ($)	706,275				
LCOE ($/kWh)	0.2116				

**Figure 16:**
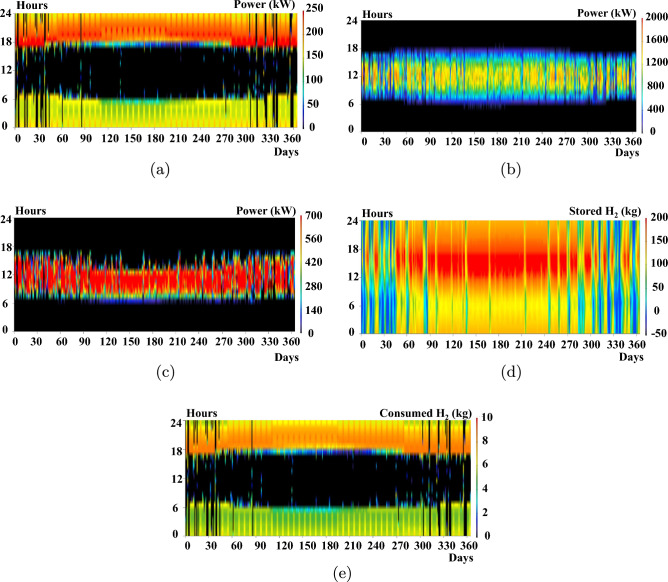
System operation profiles for 1-year simulations using HOMER.

Simulation of HOMER is conducted for 8760 h, as shown in Fig. 16, to deduce the operation profiles of FC, solar PV, wind turbine, and hydrogen tanks, respectively. This section provides an in-depth comparative analysis between standalone solar PV systems, standalone WTG, and their hybrid combination. This comparison delves into critical metrics, including system sizing capacities, LCOE, and annual energy production. The computational efficiency of different sizing methods varies considerably. When using HOMER for optimization, the time complexity can be significant, with case 2 requiring up to 14 h and case 1 taking approximately 6 h. The duration largely depends on the search space associated with each component size. In contrast, the DBM method demonstrates a much more streamlined approach. It iteratively arrives at the optimal sizing of components and their modes of operation within a broader search space in just a fraction of the time. Specifically, DBM computations can conclude in mere minutes.

**Figure 17:**
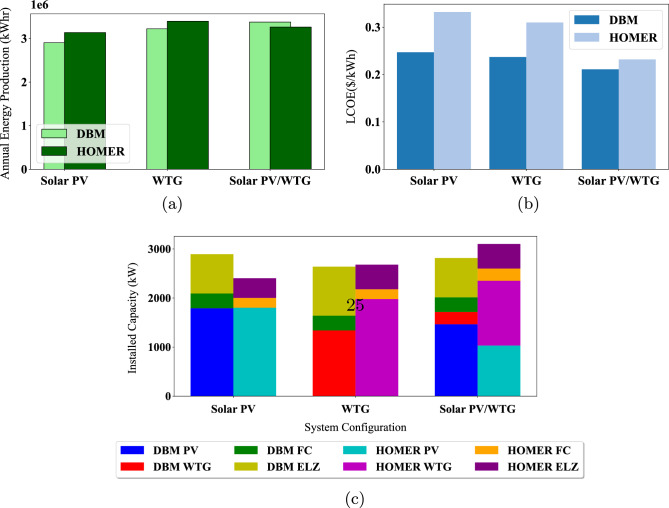
Comparative analysis of hybrid solar PV/WTG system.

Based on the comparison and cost analysis study conducted in Fig. 17, hybrid solar PV/Wind with high penetration of solar PV modules, which corresponds to the lowest LCOE, is selected for implementation in the site of Cairo International Airport to supply the community load of 250 kW peak. These figures demonstrate the close alignment in annual energy production between the two methods. Despite differing in installed capacity setups, our approach notably outperformed HOMER regarding LCOE across all configurations. This comparison uses identical model parameters and input data to ensure a fair and accurate evaluation. It is observed that HOMER favors wind energy penetration rather than solar PV. However, this penetration affects the utilization of the installed wind turbines, resulting in a lower capacity factor for the turbines.

## Conclusion

The present study has formulated a deterministic approach for the sizing optimization of hybrid energy systems, with particular attention to computational efficiency and 1-year simulations. Herein, we summarize the salient aspects and findings:

- Our proposed DBM has demonstrated a less iterative and direct approach to determining the optimal sizing of RES components for a 1-year simulation scenario. This was achieved by utilizing the area under curves method for computing energy balance on a daily base from historical datasets.
- Based on the data presented, it is evident that the DBM method consistently outperformed HOMER in achieving lower LCOE across all system configurations.
- An optimal system configuration, predominantly featuring solar PV in conjunction with wind turbines, was identified for the specific geographic conditions studied in Egypt, leading to favorable annual energy production and cost metrics.
- Comparison with HOMER software outcomes confirmed the viability of our approach, with discrepancies within an acceptable range, thus underscoring the method’s effectiveness.
- Our approach finds seasonal energy production variances due to the natural availability of solar and wind resources. The feasibility of using hydrogen tanks for energy storage has been examined, showcasing the potential for converting excess seasonal energy production into hydrogen to support future hydrogen fueling infrastructure.

Building upon our study’s deterministic optimization framework, future research will enhance the algorithm’s robustness against uncertainties in renewable energy production and demand. Efforts will focus on integrating stochastic elements and real-time data into the system, improving predictive capabilities for energy surplus management and system configuration adaptability. Additionally, we will explore the scalability of hydrogen storage solutions within the broader context of energy strategies. We aim to extend our model’s application to various geographic and climatic conditions, thus advancing hybrid energy systems’ resilience and economic viability.

## Data Availability

The datasets analyzed during the current study are available on GitHub.

## Nomenclature

*AEP*: Annual energy production (kWh)
*ANI*: Annual net income ($/year)
*AR*: Annual revenue from selling the electricity ($/year)
*CI*: Capital investment ($)
*COE*: Cost of energy ($)
*DC*: Decommissioning costs ($)
$$E^+$$: Surplus energy supplied by PV arrays
$$E^-$$: Total lack of energy which will be supplied by fuel cell and electrolyzer
$$E^{-,1}$$: Lack of energy from 12:00 a.m. till sunrise
$$E^{-,2}$$: Lack of energy from sunset till 12:00 a.m.
$$E^{D}$$: Energy demanded by load and supplied by solar PV arrays
$$E^{D,1}$$: Energy demanded by load and supplied by solar PV arrays during sunrise
$$E^{D,2}$$: Energy demanded by load and supplied by solar PV arrays during sunset
*ELZ*: Electrolyzer
*ESP*: Electricity selling price ($/kWh)
*FC*: Fuel cell
*FV*: Future value
*GT*: Irradiance power (W/m^2^)
*LAC*: Levelized annual cost ($)
*LCOE*: Levelized cost of energy ($/kWh)
*LIP*: Load interruption probability
*LSCS*: Lifespan cost of the hybrid system
*NPV*: Net present value ($)
*O &M cost*: Annual operating and maintenance cost ($/kWh)
*P*: Period of years (years)
*Pl*: Load power (kW)
*Pr*: Rated electrical power output (kW)
*PV*: Photovoltaics
$$PV^*$$: Present value of the cost analysis
*PVC*: Present value of all the costs ($)
*PVDC*: Present value of the cost of decommissioning ($)
*PVM*: Present value maintenance and repair cost
$$P_{\text {Elz}}$$: Electrolyzer power
$$P_{\text {FC}}$$: Fuel cell power
$$P_{\text {G}}$$: Generated power
$$P_{\text {PV}}$$: Solar PV power
$$P_{\text {W}}$$: Wind power
$$P_{\text {sys.,los}}$$: System losses
$$P_{\text {tank}}$$: Power stored in the tank
$$P_{h_2,\text {load}}$$: Hydrogen load power
*R*: Annual discount rate (%)
$$T_a$$: Reference temperature (25^∘^C)
$$T_m$$: Module temperature (^∘^C)
$$T_X$$: Temperature de-rating factor
$$T_e$$: End time of simulation
$$V_N$$: Rated speed (m/s)
$$V_D$$: Cut-in speed (m/s)
$$V_R$$: Cut-off speed (m/s)
$$V_V$$: Wind speed (m/s)
*WTG*: Wind turbine generator
$$\alpha$$: Power temperature coefficient for module selected (-0.331% ^∘^C)
$$E_{H_2}$$: Energy required to produce one kilogram of hydrogen
$$\eta$$: Efficiency of the system
$$\eta _{\text {Elz}}$$: Efficiency of the electrolyzer
$$\eta _{\text {FC}}$$: Efficiency of the fuel cell
$$\eta _{H_{2},\text {Tk}}$$: Efficiency of the hydrogen tank
$$HV_{H_2}$$: Heating value of $$H_2$$
$$M_{H_2}$$: Hydrogen produced in kilograms
$$\rho _{H_2}$$: Density of $$H_2$$
